# Functional intraepithelial lymphocyte changes in inflammatory bowel disease and spondyloarthritis have disease specific correlations with intestinal microbiota

**DOI:** 10.1186/s13075-018-1639-3

**Published:** 2018-07-20

**Authors:** Emilie H. Regner, Neha Ohri, Andrew Stahly, Mark E. Gerich, Blair P. Fennimore, Diana Ir, Widian K. Jubair, Carsten Görg, Janet Siebert, Charles E. Robertson, Liron Caplan, Daniel N. Frank, Kristine A. Kuhn

**Affiliations:** 1 Division of Gastroenterology, Department of Medicine, University of Colorado School of Medicine, Aurora, CO USA; 2 Division of Rheumatology, Department of Medicine, University of Colorado School of Medicine, Aurora, CO USA; 3 Division of Infectious Disease, Department of Medicine, University of Colorado School of Medicine, Aurora, CO USA; 4 Mucosal Inflammation Program, University of Colorado School of Medicine, Aurora, CO USA; 5 Denver Veterans Affairs Medical Center (Denver VAMC), Denver, CO USA; 6 Computational Bioscience Program, University of Colorado School of Medicine, Aurora, CO USA

**Keywords:** Inflammatory bowel disease, Ulcerative colitis, Crohn’s disease, Spondyloarthritis, Intraepithelial lymphocytes, Microbiome

## Abstract

**Background:**

Dysbiosis occurs in spondyloarthritis (SpA) and inflammatory bowel disease (IBD), which is subdivided into Crohn’s disease (CD) and ulcerative colitis (UC). The immunologic consequences of alterations in microbiota, however, have not been defined. Intraepithelial lymphocytes (IELs) are T cells within the intestinal epithelium that are in close contact with bacteria and are likely to be modulated by changes in microbiota. We examined differences in human gut-associated bacteria and tested correlation with functional changes in IELs in patients with axial SpA (axSpA), CD, or UC, and in controls.

**Methods:**

We conducted a case-control study to evaluate IELs from pinch biopsies of grossly normal colonic tissue from subjects with biopsy-proven CD or UC, axSpA fulfilling Assessment of SpondyloArthritis International Society (ASAS) criteria and from controls during endoscopy. IELs were harvested and characterized by flow cytometry for cell surface markers. Secreted cytokines were measured by ELISA. Microbiome analysis was by 16S rRNA gene sequencing from rectal swabs. Statistical analyses were performed with the Kruskal-Wallis and Spearman’s rank tests.

**Results:**

The total number of IELs was significantly decreased in subjects with axSpA compared to those with IBD and controls, likely due to a decrease in TCRβ+ IELs. We found strong, significant negative correlation between peripheral lymphocyte count and IEL number. IELs secreted significantly increased IL-1β in patients with UC, significantly increased IL-17A and IFN-γ in patients with CD, and significantly increased TNF-α in patients with CD and axSpA as compared to other cohorts. For each disease subtype, IELs and IEL-produced cytokines were positively and negatively correlated with the relative abundance of multiple bacterial taxa.

**Conclusions:**

Our data indicate differences in IEL function among subjects with axSpA, CD, and UC compared to healthy controls. We propose that the observed correlation between altered microbiota and IEL function in these populations are relevant to the pathogenesis of axSpA and IBD, and discuss possible mechanisms.

**Trial registration:**

ClinicalTrials.gov, NCT02389075. Registered on 17 March 2015.

**Electronic supplementary material:**

The online version of this article (10.1186/s13075-018-1639-3) contains supplementary material, which is available to authorized users.

## Background

Spondyloarthritis (SpA), characterized by inflammation of vertebral and peripheral joints sometimes progressing to fusion of the sacroiliac joints, affects up to 1% of the population and includes the diseases ankylosing spondylitis (AS), psoriatic arthritis, reactive arthritis, and inflammatory bowel disease (IBD)-related SpA. While symptoms start in the second and third decade of life, there are no disease-specific biomarkers, often causing delays in diagnosis resulting in increased functional impairment and disability [1]. IBD, which is significantly associated with SpA, is characterized by relapsing and remitting intestinal inflammation and can be subdivided into Crohn’s disease (CD) and ulcerative colitis (UC). Affecting up to 0.5% of the population, IBD also lacks a disease-specific biomarker and results in substantial morbidity [2]. The clinical overlap of SpA with IBD, and observations of reactive arthritis triggered by intestinal pathogens, suggest a shared pathogenesis between the two diseases, which is likely driven by genetic susceptibility and an environmental trigger provided by intestinal microbes [3].

The most frequently cited genetic susceptibility for SpA is conferred with major histocompatibility complex (MHC) class I allele HLA-B27, which is found in 85% of individuals with axial SpA (axSpA). In non-axial SpA the presence of HLA-B27 positivity is much lower. For example, in those with IBD-associated SpA, 63% are HLA-B27+ [4]. HLA-B27 positivity is thought to result in altered recognition and handling of bacterial antigens [5, 6]. Some evidence also links IL-23 receptor variants to susceptibility risk for both axSpA and IBD. IL-23 acts as a regulator of cellular immunity by promoting a T helper (Th)17 response [7], which develops at the intestinal mucosa in the presence of commensal bacteria [8]. Thus, there appears to be an association between genetic susceptibility, intestinal microbiota, and the mucosal immune response in the pathophysiology of both axSpA and IBD.

In addition to association with genes involved in altered recognition and handling of bacterial antigens, both axSpA [9–11] and IBD [12, 13] have a well-known association with dysbiosis (alteration in microbiota)—a finding that extends to newly diagnosed and untreated patients [14]. However, the effect of dysbiosis on disease onset, progression, and recurrence is unclear [12]. It is possible that dysbiosis-mediated alteration in mucosal immune cell function plays a key role in disease pathogenesis.

We have previously shown that the function of colonic intraepithelial lymphocytes (IELs) is altered by dysbiosis in mice [15]. The epithelium is the most superficial layer of the alimentary tract and as such, would be expected to be affected to the greatest degree by alterations in the microbiome. Thus, IELs serve as prime targets to study how changes in resident bacteria influence immune response. These cells have the contradictory role of tolerating colonization by resident bacteria while still mounting a prompt and robust immune response against invading pathogens [16]. Moreover, intraepithelial lymphocytosis has a well-known association with other immune-mediated diseases of the gut including lymphocytic colitis, celiac disease, autoimmune enteropathy, and allergic colitis [17, 18]. Unlike those in healthy individuals, IELs from patients with IBD do not downregulate proliferative responses of primed allogeneic peripheral blood mononuclear cells on re-challenge with antigens [19]. Thus, dysregulation of IELs may provide new insights into the influence the microbiome has on systemic and mucosal immunity.

In this study, we hypothesized that alterations in microbiota in axSpA and IBD would be associated with functional changes in colonic IELs. Specifically, we examined total number and type of IELs and individual cytokine secretion by IELs, and we completed an exploratory analysis of microbiome interaction with IELs and cytokine secretion. We found unique patterns of IEL phenotype, cytokine expression, and cytokine-microbiome interactions by disease state that begin to elucidate how the microbiome affects immunity.

## Methods

### Study population and design

This study utilized a case-control study design. Control participants were recruited from the routine endoscopy schedule at the University of Colorado Hospital in Aurora, Colorado from March 2015 to January 2017; these subjects were undergoing colonoscopy for colon cancer screening (at age 50 years or younger if a family history of colon cancer is present) or due to changes in bowel habits. Subjects were included only if they were negative for malignancy and inflammation on biopsies. Patients with IBD (cases) were recruited if undergoing disease activity assessment or colon cancer or dysplasia screening; only those negative for cancer or dysplasia were included in the study. Patients with AxSpA (cases) were recruited to undergo an elective flexible sigmoidoscopy (*n* = 4) or underwent colonoscopy due to symptoms of a change in bowel habits (*n* = 2). Those with change in bowel habits were only included when the endoscopic exam and colonic and ileal biopsies excluded IBD. Fifteen biopsies were taken from endoscopically non-inflamed portions of the left colon (descending, sigmoid, and rectum) so as to avoid the confounding effect of inflammation, as IEL number has previously been shown to increase in active colitis [20]. Patients were eligible if they had biopsy-proven IBD or axSpA fulfilling the 2009 Assessment of SpondyloArthritis International Society (ASAS) criteria [21]; individuals with an overlap of both IBD and SpA were excluded, to allow for clear discrimination between cohorts. Controls were eligible if they did not have IBD or SpA and did not meet the exclusion criteria. Exclusion criteria for all groups included pregnancy, use of antibiotics within the past 14 days, current colon cancer, celiac disease, diagnosis of any rheumatologic disease (except in the axSpA group in which only a diagnosis of SpA was permitted), chemotherapy or radiation therapy for any malignancy within the past year, daily use of aspirin or non-steroidal anti-inflammatory drugs (NSAIDs) with inability to hold the drug 7 days before and after the procedure, use of anticoagulation, HIV, *Clostridium difficile* infection within the past 3 months, or evidence of inflammatory spinal or axial arthritis (post-inflammatory changes on radiographs or a diagnosis of sacroiliitis) based on chart review (except in the axSpA group).

Information about demographic characteristics, medical history, disease history, and family history were abstracted from charts. All subjects completed a questionnaire inquiring about dietary habits such as meat and fish consumption, fruit and vegetable consumption, and whole grain consumption. Patients (cases) completed questionnaires to obtain the Harvey Bradshaw Index (HBI) in CD, the Simple Clinical Colitis Activity Index (SCCAI) in UC, and the Bath Ankylosing Spondylitis Disease Activity Index (BASDAI) in axSpA. Blood was collected to measure C-reactive protein (CRP) and white blood cell count (WBC) if not measured within the past 30 days, and to test HLA-B27 status if not already performed for routine clinical care. Fecal samples were collected by cotton swab of the rectum and stored at ˗ 80 °C until analysis. During endoscopy, 15 pinch biopsies of colonic mucosa were taken from endoscopically normal-appearing colonic tissue, combined in phosphate-buffered saline (PBS) on ice, and taken to the laboratory for further processing.

All clinical investigations were conducted according to the principles expressed in the Declaration of Helsinki. The study protocol was approved by the Colorado Multiple Institutional Review Board. All patients provided written informed consent and authorization for release of personal health information. An independent safety officer was assigned and met every 12 months with the investigators to conduct safety reviews.

### Analysis of IELs

IELs were harvested from tissue by vortexing the 15 pinch biopsies together in 10 mL of PBS with 1 mM EDTA for 10 min at room temperature. They were filtered through a 70-μm cell filter, centrifuged, and resuspended in 1 mL of PBS with 5% fetal calf serum. T cells were counted using a hemocytometer then divided: 200 μL for flow cytometry and 800 μl for magnetic sorting using a modified human T cell isolation kit (StemCell Technologies, Vancouver, BC, Canada). The isolated human epithelial cells from pinch biopsies were mixed with 10 μl of human T cell enrichment cocktail (StemCell Technologies) and 2 μL biotinylated anti-CD236 (1B7, eBiosciences, San Diego, CA, USA) for 10 min at room temperature. The remaining steps of the kit protocol were then followed to complete magnetic separation. The sorted cells were again counted to corroborate with our presorted cell counts in which IELs were quantified using flow cytometry data. The flow cytometry percentages with presort counts were used to calculate IEL numbers except in the few cases (four individuals) in which the flow cytometry data were of poor quality. In those cases, the post-sort counts were used for total IEL numbers, but further T cell subsets could not be quantified.

Post-sorted IELs were mitogen-stimulated with 10 ng/mL of phorbol 12-myristate 13-acetate (PMA) and 1 ng/mL ionomycin overnight. Cytokines IL-1β, IL-6, IL-10, IL-17A, interferon (IFN)-γ, and TNF-α in the culture supernatants were measured by ELISA (Meso Scale Discovery, Rockville, MD, USA).

For flow cytometry, cells were stained for surface markers CD3 (UCHT1), CD45 (HI30), CD4 (RPA-T4), CD8α (RPA-T8), CD8β (SIDI8BEE), CD44 (IM7), CD103 (B-Ly7), TCRβ (WT31), TCRγδ (B1.1), and a viability dye (Ghost Dye™ Violet 510, Tonbo Biosciences, San Diego, CA, USA) and then fixed in 4% paraformaldehyde in PBS. Samples were run on a LSR II (Becton, Dickinson and Company Biosciences, San Jose, CA, USA) and data analyzed with FlowJo version 10.1 (Treestar, San Carlos, CA, USA).

### Microbiome analysis

DNA from feces of cases and controls was extracted using a commercial kit (UltraPure Fecal DNA, MO BIO Inc., Carlsbad, CA, USA). Bacterial profiles were determined by broad-range amplification and sequence analysis of 16S rRNA genes following our previously described methods [22–25]. In brief, amplicons were generated using barcoded primers [26] that target approximately 340 base pairs of the V3 V4 variable region of the 16S rRNA gene. Illumina paired-end sequencing was performed on the Miseq platform with versions v2.4 of the Miseq Control Software and of MiSeq Reporter, using a 600 cycle version 3 reagent kit.

Illumina Miseq paired-end reads were aligned to human reference genome hg19 with bowtie2 and matching sequences discarded [27, 28]. As previously described, the remaining non-human paired-end sequences were demultiplexed [24], assembled [29, 30], trimmed (moving window of five nucleotides until average quality was met or exceeded 20), and chimera-checked with Uchime (usearch6.0.203_i86linux32) [31] using the Schloss [32] Silva reference sequences. Trimmed sequences with more than 1 ambiguity or shorter than 250 nucleotides were discarded. Assembled sequences were aligned and classified with SINA (1.3.0-r23838) [33] using the 418,497 bacterial sequences in Silva 115NR99 [34] as reference configured to yield the Silva taxonomy. Operational taxonomic units (OTUs) were produced by clustering sequences with identical taxonomic assignments. This process generated ~ 6,000,000 high-quality 16S sequences with Goods coverage score ≥ 99% for all samples. Relative abundances for each subject were calculated by dividing the sequence count for each OTU by the total number of 16S rRNA sequences for the subject. The software package Explicet (v2.10.5, www.explicet.org) [35] was used for visualization of the data.

### Statistical analysis

A total sample size of 24 was determined to have 80% power to detect an effect size of 0.75 across four groups using one-way analysis of variance (ANOVA) with a two-tailed significance of 0.05 for the primary outcome of differences in IELs between study cohorts. Variables were tested for normality using the Shapiro-Wilk test; those data not normally distributed are presented in the figures using a logarithmic y-axis. Patient demographic comparisons were made using ANOVA or Fisher’s exact test to compare patients with CD, UC, and axSpA, and controls, except for medications, which were compared in the CD, UC, and axSpA groups only. The Kruskal-Wallis test with Dunn’s post-hoc analysis for pairwise comparisons of significant groups was used to evaluate differences in IEL phenotype and cytokine production. Linear regression was used to test correlation between IEL characteristics and continuous variables including CRP, WBC, duration of disease, disease activity scores, and age. Fisher’s exact test was utilized to test associations between IEL secretion and categorical variables such as sex, family history of autoimmunity, smoking history, diet, and TNF inhibitor (TNFi) use. The Kruskal-Wallis test was used to compare relative abundance of OTUs between study groups. Spearman’s rank correlation was tested between OTUs and IEL changes that were found to be statistically significantly different between groups. Permutation-based multiple analysis of variance (PERMANOVA) was conducted using the vegan R package (Bray-Curtis Method, 10,000 permutations).

Community diversity was estimated by both the Shannon H index and Simpson inverse diversity indices. Chao1 was used to measure microbial richness and Good’s coverage was used to confirm the adequacy of sequencing coverage. All tests of significance were two-sided and a *p* value < 0.05 was considered statistically significant. Statistical analyses were conducted with the R and SPSS (version 22; IBM, Armonk, NY, USA) statistical packages, while graphics were created using SPSS, GraphPad version 8.2 (GraphPad Software, La Jolla, CA, USA), and Excel Office Professional Plus 2013 (Microsoft, Redmond, WA, USA).

## Results

### Baseline characteristics

A total of 38 subjects (15 controls, 10 patients with CD, 7 patients with UC, and 6 patients with axSpA) were recruited (Table 1). There were no significant differences in baseline basic demographic features (sex, ethnicity, smoking status, family history of autoimmunity). More patients with CD (50%) or axSpA (83%) were on TNF inhibitors (TNFi) compared to patients with UC (0%, *p* < 0.01). As expected, patients with axSpA (83%) were more likely to be HLA-B27-positive than patients with IBD (20%) or controls (0%, *p* = 0.0001). Patients with axSpA were noted to have significantly higher leukocyte counts than patients with IBD and controls (*p* = 0.02), although the increased leukocyte count was not outside the normal range determined by the clinical laboratory. Overall, subjects with IBD had long-established disease and low disease activity at the time of their endoscopy. Patients with axSpA also had long-established disease, moderate disease activity, and more TNFi use at the time of their endoscopy.

**Table 1:** Demographic and clinical characteristics of study subjects

	Controls (*N* = 15)	CD (*N* = 10)	UC (*N* = 7)	axSpA (*N* = 6)	*p* value
Age in years	48.4 (9.7)	40.5 (15.2)	39.2 (12.4)	34.4 (3.3)	0.06
Female	9 (60%)	7 (70%)	3 (43%)	1 (17%)	0.18
Non-Hispanic white	15 (100%)	9 (90%)	6 (86%)	6 (100%)	0.29
Former smoker	4 (27%)	5 (50%)	1 (14%)	3 (50%)	0.34
Family history of autoimmunity	6 (40%)	3 (30%)	1 (14%)	2 (33%)	0.53
Disease duration in months	–	127.5 (107.9)	171.7 (153.9)	78.5 (58.1)	0.36
HLA-B27 positivity	0	0	1 (14%)	5 (83%)	**0.0001** ^**a**^
WBC (10^9^ cells/L)	7.2 (2.2)	6.6 (2.2)	5.5 (3.2)	9.5 (2.3)	**0.04**
Absolute lymphocytes (10^9^ cells/L)	1.9 (0.1)	1.7 (0.2)	0.9 (0.1)	2.6 (0.3)	**0.004**
CRP (mg/L)	2.5 (2.5)	4.2 (3.0)	1.7 (1.7)	5.6 (12.5)	0.5
TNF inhibitor usage	–	5 (50%)	0 (0%)	5 (83%)	**< 0.01**
Prednisone usage	–	2 (20%)	0 (0%)	1 (17%)	0.5
Harvey Bradshaw Index	–	1.9 (0.526)		–	–
Simple Clinical Colitis Activity Index	–	–	0.857 (0.553)	–	–
Bath AS Disease Activity Index	–	–	–	3.96 (1.01)	–

Data are mean (SD) or number (%); *p* value is based on Fisher’s exact test for categorical variables and on one-way analysis of variance for continuous variables. ^a^Bold text indicates significant *p*-values

*HC* healthy controls, *CD* Crohn’s disease, *UC* ulcerative colitis, *axSpA* axial spondyloarthritis, *CR*P C-reactive protein, *AS* ankylosing spondylitis

### IEL populations

**Fig. 1:**
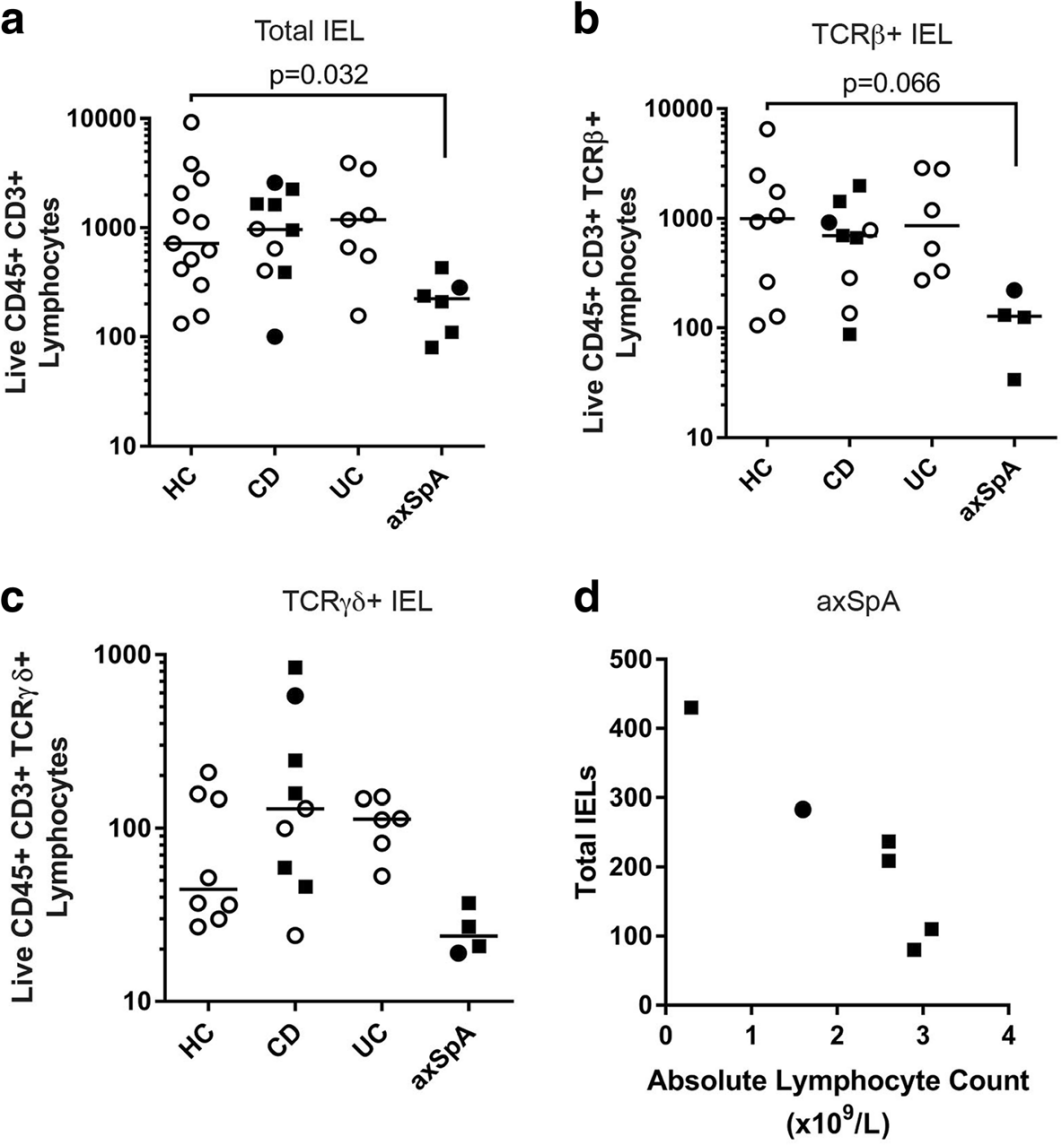
Colonic intraepithelial lymphocytes (IELs) are significantly decreased in individuals with axial spondyloarthritis (axSpA) and inversely correlate with peripheral blood lymphocyte counts. IELs were obtained from colonic mucosal biopsies as described in “Methods” and evaluated by flow cytometry for the absolute number of total IELs (**a**), T cell receptor (TCR)β+ IELs (**b**), and TCRγδ+ IELs (**c**). Each dot represents an individual within the study group identified on the x-axis. A solid square indicates subjects on a TNF inhibitor, a solid dot indicates the subject was taking steroids, and an open dot indicates the subject was taking neither. Bars are the mean ± SEM. Statistical significance was determined using the Kruskal-Wallis test with Dunn’s post-hoc analysis. **d** Total IELs (y-axis) in each individual with axSpA were compared to the absolute lymphocyte count (x-axis) and the Pearson’s correlation coefficient was calculated (σ = − 0.94, *R*^2^ = 0.89, *p* = 0.0047). HC, healthy controls; CD, Crohn’s disease; UC, ulcerative colitis

The total number of IELs was significantly decreased in subjects with axSpA compared to those with IBD and controls (*p* = 0.03; Fig. 1a). When analyzed by T cell subsets, a decrease in T cell receptor (TCR)β+ IELs likely accounted for this difference (*p* = 0.06; Fig. 1b). There were trends toward reduced TCRβ+ subsets of CD4+ and CD8aa+ IELs (*p* = 0.074 and *p* = 0.030, respectively; Additional file 1: Figure S1A), but no significant differences between cohorts in total TCRγδ+ IELs (Fig. 1c) or in CD4+, CD4-CD8-, CD8αα+, and CD8αβ+ subsets of TCRγδ+ IELs (Additional file 1: Figure S1B). Nor were there significant differences between cohorts in the presence of cell markers CD44+, CD103+ (integrin αE, which mediates retention of the IEL at the epithelial surface [36]), or IL-23R (Additional file 1: Figure S1C-E). As the number of IELs could be influenced by the systemic level of lymphocytes, which were significantly higher in subjects with axSpA, we tested for correlation between IELs and peripheral blood absolute lymphocyte count in those with axSpA. To our surprise, we found strong, significant negative correlation between the peripheral lymphocyte count and IEL number in subjects with axSpA (*ρ* = − 0.94, *R*^2^ = 0.89, *p* = 0.0047; Fig. 1d). We did not, however, observe significant correlation between IEL number and disease activity in any of our cohorts (Additional file 2: Table S1).

**Fig. 2:**
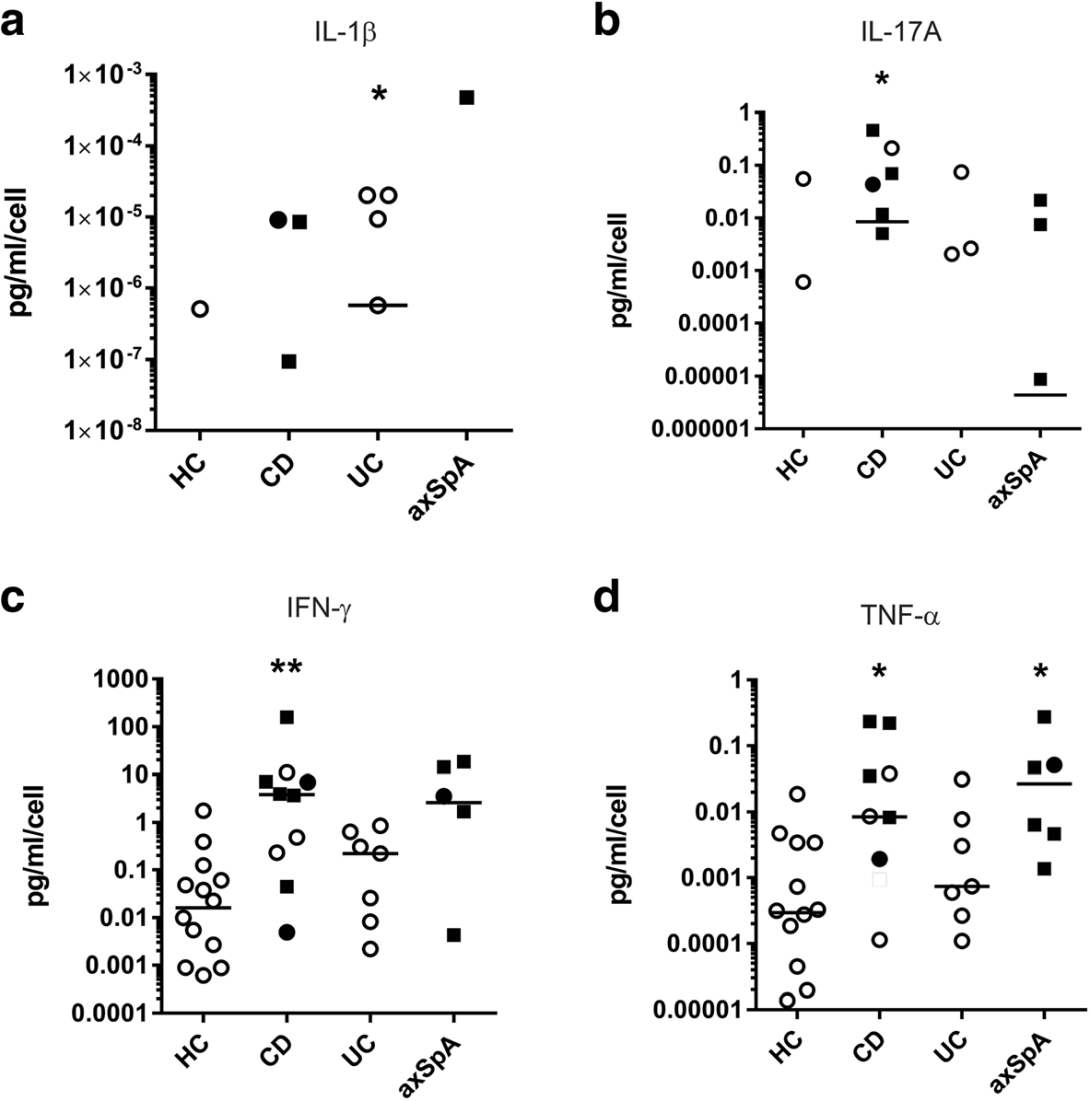
Cytokines produced by intraepithelial lymphocytes (IELs) are altered in individuals with inflammatory bowel disease (IBD) and axial spondyloarthritis (axSpA). Colonic IELs were mitogen-stimulated overnight and secreted cytokines measured by ELISA. **a** IL-1β, **b** IL-17A, **c** interferon (IFN)-γ, and **d** TNF-α production in pg/ml was normalized to the number of IELs collected from each subject. Each dot represents a case/control analyzed. A solid square indicates subjects on a TNF inhibitor, a solid dot indicates the subject was taking steroids, and an open dot indicates the subject was taking neither. Subjects with undetectable cytokine levels are absent from the graphs due to the logarithmic scale as are means that fall below the axis range. Statistical differences were identified by the Kruskal-Wallis test with Dunn’s post-hoc analysis for pairwise differences: *p < 0.05 and **p < 0.01. HC, healthy controls; CD, Crohn’s disease; UC, ulcerative colitis; axSpA, axial spondyloarthritis

Because we hypothesized that subjects with axSpA and IBD would exhibit functional changes in IELs compared to control subjects, we evaluated individual cytokine secretion by IELs obtained from biopsy tissue. A few subjects had dramatically elevated levels of certain cytokines but these were not limited to the same subjects. IELs secreted significantly increased IL-1β in patients with UC as compared to other cohorts (Fig. 2a), significantly increased IL-17A and IFN-γ in patients with CD as compared to other cohorts (Fig. 2b, c), and significantly increased TNF-α in patients with CD and axSpA as compared to other cohorts (Fig. 2d). The data are shown on a logarithmic axis to demonstrate the range of cytokine values. No significant differences in IEL secretion of IL-6, IL-10, or IL-22 was detected between cohorts (Additional file 1: Figure S2).

Sex, age, race, length of disease, disease activity scores, CRP, and family history of autoimmune disease was not associated with the presence or absence of individual cytokine secretion when analyzed using linear regression (Additional file 2: Table S2). Smoking history was negatively associated with secretion of TNF-α (*p* = 0.008), and TNFi use was associated with secretion of TNF-α and IFN-γ (*p* = 0.001 and *p* = 0.04, respectively). Kruskal-Wallis analysis did not identify any significant association between cytokine levels and intake of red meat, fish, whole grains, fruits, or vegetables (Additional file 2: Table S2).

### Microbiome associations

Bacterial profiling of subjects’ fecal material was of sufficient depth as indicated by Good’s coverage > 99% for all subjects. No difference in richness (Chao1) was noted among the three groups (Additional file 1: Figure S3A). Analysis of diversity indices showed no difference in community complexity, as measured by the mean Shannon H index (Additional file 1: Figure S3B), or in community evenness (Additional file 1: Figure S3C).

**Fig. 3:**
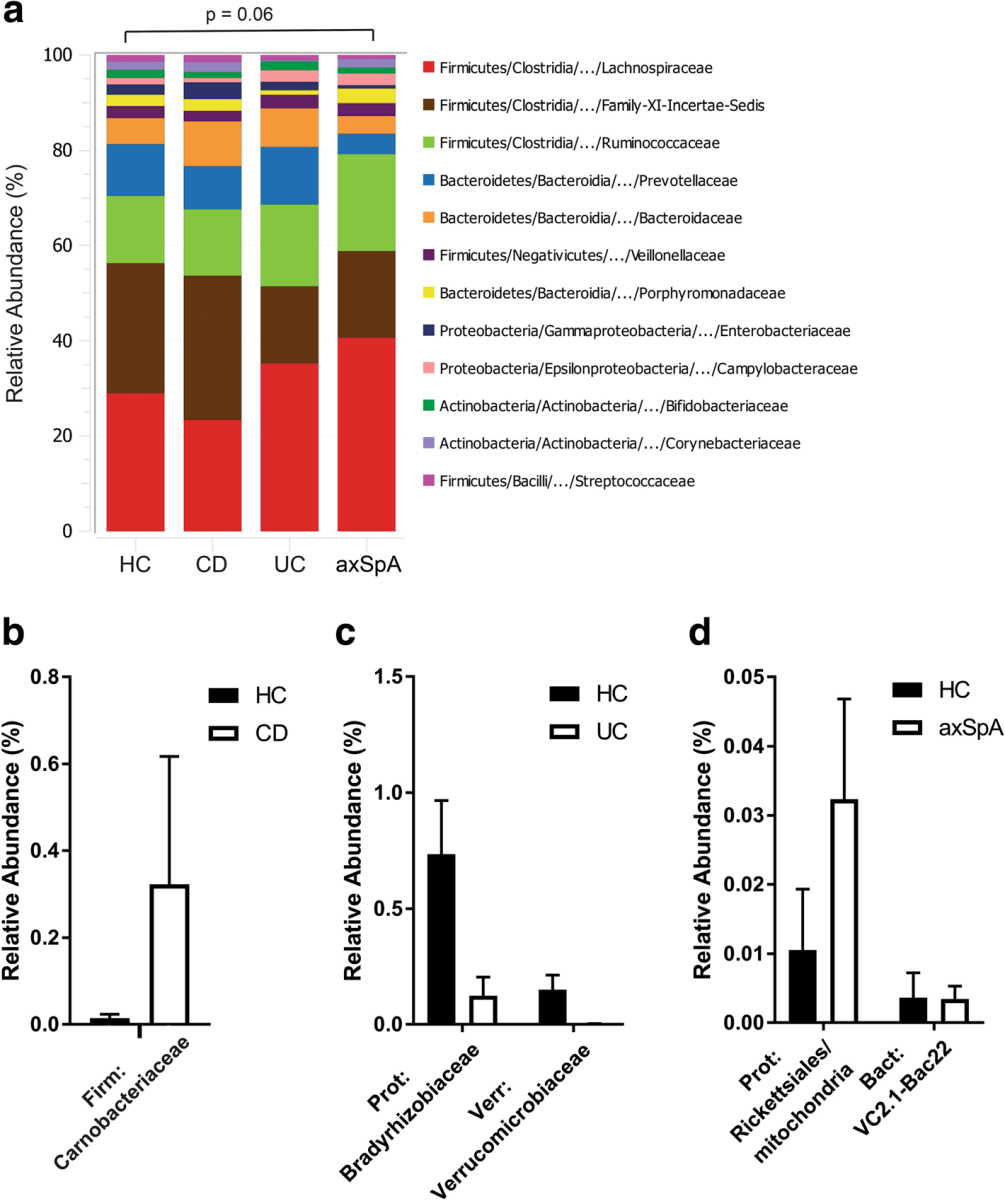
Microbial community comparisons in individuals with inflammatory bowel disease (IBD) and axial spondyloarthritis (axSpA). Bacterial DNA from fecal swabs from patients (cases) and controls were sequenced for 16S rRNA and analyzed. **a** The percent abundance of the top operational taxonomic unit (OTU) families were compared across subject groups. Differences in the overall composition of microbial communities were determined by permutation-based multiple analysis of variance. **b**–**d** Pairwise comparisons of OTUs between disease states and controls were performed using the Wilcoxon rank-sum test. The mean relative abundance ± SEM for each OTU that was statistically significant (*p* < 0.05) is shown for Crohn’s disease (CD) (**b**), ulcerative colitis (UC) (**c**), and axSpA (**d**). HC, healthy controls

Our study was primarily powered to evaluate differences in IEL phenotypes across groups. Nevertheless, our exploratory analysis identified appreciable differences in overall microbiota composition between healthy controls and subjects with axSpA (PERMANOVA *p* = 0.06; Fig. 3a). In pairwise comparisons between healthy controls and patients with CD, UC, and axSpA we identified few significant differences as defined by *p* < 0.05 analyzed by the Wilcoxon rank-sum test. In CD there was increased relative abundance of *Carnobacteriaceae* (Fig. 3b). In healthy controls, as compared to patients with UC, the relative abundance of *Bradyrhizobiaceae* was increased (Fig. 3c). In patients with axSpA as compared to healthy controls, the relative abundance of *Rickettsiales* was increased (Fig. 3d).

We next evaluated how IELs related to specific bacteria in the setting of disease. For each subject group, total IEL number or IEL-produced cytokine level (using only those cytokines that were significantly different between subject groups) was correlated with the relative abundance of each OTU.

**Fig. 4:**
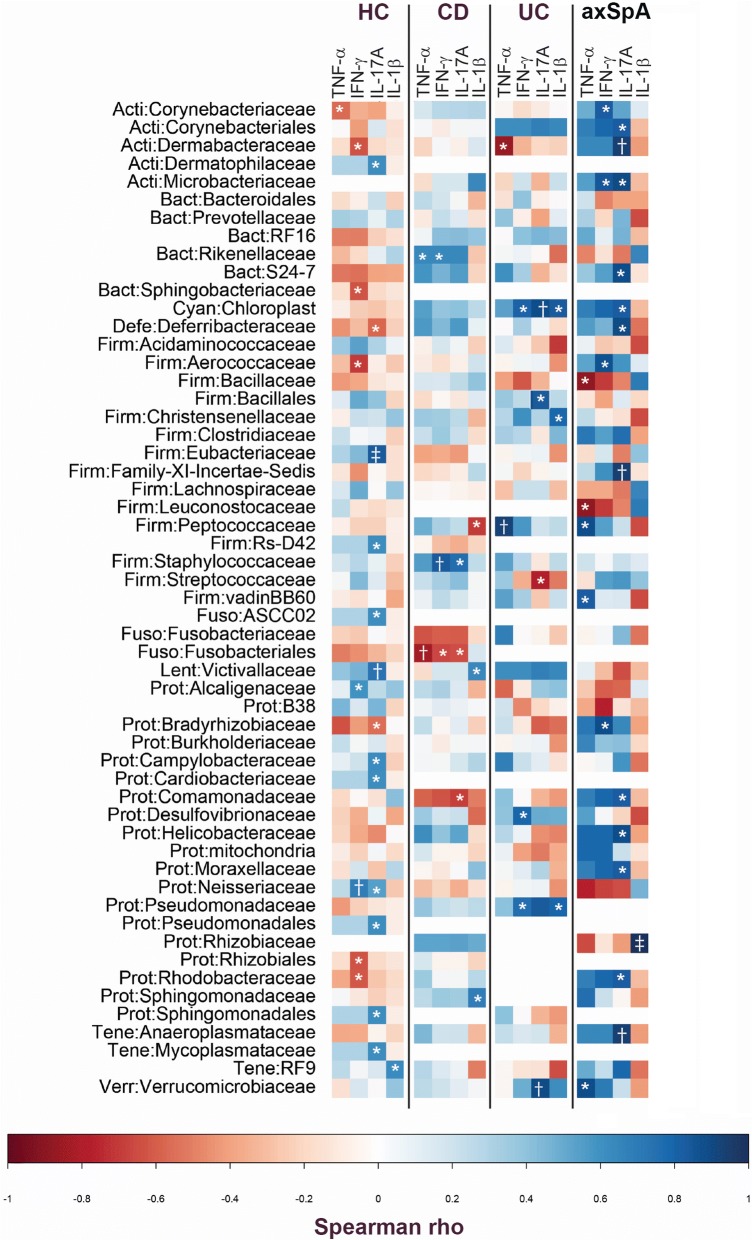
There was disease-specific correlation between microbiota and intraepithelial lymphocyte (IEL) production of cytokines. Correlation between the operational taxonomic unit (out) relative abundance and the IEL-produced cytokine value in each case/control group was tested using Spearman’s rank test. The data are shown as heatmaps, with the color of each correlation test corresponding to the Spearman rho value: *p < 0.05, ^†^*p* < 0.01, and ^‡^*p* < 0.001. HC, healthy controls; CD, Crohn’s disease; UC, ulcerative colitis; axSpA, axial spondyloarthritis

Within the healthy control group, we identified statistically significant positive correlation between *Eubacteriaceae*, *Victivallaceae*, *Campylobacteraceae*, *Cardiobacteriaceae*, *Neisseriaceae*, and *Pseudomonadales* and IL-17A and between *Alcaligenaceae* and *Neiseriaceae* and IFN-γ (Fig. 4). Also in the healthy control group, we identified statistically significant negative correlation between the abundances of *Deferribacteraceae* and IL-17A; between *Corynebacteriaceae* and TNF-α; and between *Dermabacteraceae*, *Sphingobacteriaceae*, *Aerococcaceae*, *Rhizobiales*, and *Rhodobacteraceae* and IFN-γ (Fig. 4). Within the CD group, there was statistically significant positive correlation between the abundances of *Victivallaceaea* and *Sphingomonadaceae* and IL-1β; between *Staphylococcaceaea* and IL-17A; between *Rikenellaceae* and TNF-α; and between *Staphylococcaceaea* and *Rikenellaceae* and IFN-γ (Fig. 4). Also in the CD group, we identified statistically significant negative correlation between the abundances of *Peptococcaceae* and IL-1β; between *Fusobacteriales* and *Comamonadaceae* and IL-17A; between *Fusobacteriales* and TNF-α; and between *Fusobacteriales* and IFN-γ (Fig. 4).

In the group of individuals with UC, we identified statistically significant positive correlation between the abundances of *Cynanobacteria*, *Christensenellaceae*, and *Pseudomonadaceae* and IL-1β; between *Cynanobacteria*, *Bacillales*, and *Verrucomicrobiaceae* and IL-17A; between *Peptococcaceae* and TNF-α; and between *Cynanobacteria*, *Desulfovibrionaceae*, and *Pseudomonadaceae* and IFN-γ. Also in this group, there was statistically significant negative correlation between the abundances of *Streptococcoaceae* and IL-17A and between *Dermabacteraceae* and TNF-α (Fig. 4).

In axSpA we identified statistically significant positive correlation between the abundances of *Rhizobiaceae* and IL-1β; between *Corynebacteriales*, *Dermabacteraceae*, *Microbacteriaceae*, *Deferribacteraceae*, *Cynanobacteria*, *Comamonadaceae*, *Helicobacteraceae*, *Moraxellaceae*, *Rhodobacteraceae*, and *Anaeroplasmataceae* and IL-17A; between *Peptococcaceae* and *Verrucomicrobiaceae* and TNF-α; and between *Corynebacteriaceae*, *Microbacteriaceae*, *Aerococcaceae*, and *Bradyrhizobiaceae* and IFN-γ. In axSpA, we also identified statistically significant negative correlation between the abundances of *Bacillaceae* and *Leuconostocaceae* and TNF-α (Fig. 4).

Because we identified strong negative correlation between peripheral lymphocyte count and IELs that may suggest mucosal lymphocyte trafficking in the circulation, we queried if this could be driven by specific microbiota. When IEL numbers were compared to the relative abundance of family-level OTUs, we identified only strong negative correlation between IEL number and *Dermatophilaceae*, *Prevotellaceae*, RF16, *Deferribacteraceae*, *Clostridiaceae*, *Anaeroplasmataceae*, and RF9 (Table 2), suggesting that if IELs are trafficking, they may be stimulated by these bacteria.

**Table 2:** Significant correlation between IEL number and OTU in individuals with axSpA

	Spearman’s Rho	*p* value
*Bacteroidales: Prevotellaceae*	−0.8857	0.0188
*Firmicutes: Clostridiaceae*	−0.8286	0.0416
*Deferribacteres: Deferribacteraceae*	−0.9276	0.0077
*Tenericutes: Anaeroplasmataceae*	−0.8804	0.0206
*Actinobacteria: Dermabacteraceae*	−0.8804	0.0206
*Tenericutes: RF9*	−0.9856	0.0003
*Bacteroidales: RF16*	−0.8452	0.0341
*Firmicutes: Eubacteriaceae*	0.8697	0.0244

Intraepithelial lymphocyte (IEL) numbers and relative abundance of bacterial operational taxonomic units (OTUs) were compared by Spearman’s rank correlation coefficient, which is reported alongside the *p* value for the most significant results

## Discussion

In this study we hypothesized that IEL function would vary by disease type and would be modulated by microbial milieu. Herein, we show for the first time that colonic IELs undergo functional changes in SpA and IBD. Furthermore, we suggest that changes in the microbiome may drive the functional changes observed in colonic IELs, specifically through interactions affecting cytokine secretion as demonstrated by the unique cytokine-microbiome interactions within each disease.

Our results are in agreement with prior studies that suggested no difference in absolute IEL number in subjects with IBD as compared to healthy controls. However, one of our most interesting findings is that total IEL count is significantly reduced in axSpA. Prior studies document that IELs function to maintain epithelial homeostasis and that IELs promote epithelial barrier function through expressed cytokines, such as IL-6, in response to commensal bacteria [15, 37]. A decrease in IEL count in axSpA would be consistent with the frequently observed barrier dysfunction associated with subclinical colitis in many patients with axSpA [6].

Furthermore, we identified strong, significant, negative correlation between peripheral lymphocyte count and colonic IEL count in patients with axSpA, which may suggest IEL trafficking between blood and colon compartments in this disease, although our data do not confirm this hypothesis. It has been previously established that in enterogenic SpA, activated T cells originating in inflamed intestinal mucosa develop non-gut-specific adhesion ligands, including synovial ligands, leading to homing of activated T cells from gut into synovium and perpetuating joint inflammation [38, 39]. Unfortunately, our study was not designed to evaluate peripheral blood phenotypes in parallel with mucosal phenotypes. Certainly, additional studies are warranted to validate our findings and support our suggestion that IELs traffic systemically in axSpA.

Because we hypothesized that subjects with axSpA and IBD would exhibit functional changes in IELs, we examined associations between disease type and IEL-produced cytokine quantity and type. Our findings within each disease cohort suggest that IELs may contribute to disease-specific pathology. For example, IELs isolated from colonic tissue secreted significantly increased IL-1β in patients with UC as compared to other cohorts. Since expression of the pro-inflammatory cytokine IL-1β has previously been observed to be increased in mucosal cells from patients with both UC and CD [40], IELs may be an additional local contributor to disease pathogenesis. Colonic IELs secreted significantly increased IL-17A and IFN-γ within the CD cohort compared to other cohorts. Previously published findings corroborate these findings by reporting increased gene expression of IL-17A and IFN-γ in colonic biopsies taken from patients with UC and CD compared to healthy controls [41]. Finally, in patients with CD and axSpA compared to other cohorts, colonic IELs secreted significantly increased TNF-α, which is a well-established pro-inflammatory mediator in both diseases. Thus, while IEL numbers may not be significantly different in individuals with IBD, there are IEL functional changes that may contribute to local inflammation. Because we found no relationship between age, sex, duration of disease, disease activity index, or CRP and IEL abundance or cytokine production, we suggest that alterations in microbiota found in the disease states may influence cytokine production by IELs. Of note, we did find a negative association between smoking and TNFα secretion. This finding is consistent with prior studies as it has previously been shown in rat models that TNFα expression in colonic tissue increases with exposure to cigarette smoke in a dose-dependent fashion [42]. We also noted that TNFi use was associated with increased TNFα production by IELs. TNFα production is likely a reflection of disease activity and TNFi use is presumably a marker of more active disease.

Although not powered for microbiome comparisons, our pilot microbiome sequencing data were consistent with prior studies. Specifically, we noted increased relative abundance of *Ruminococcus* in patients with SpA as compared to healthy controls, which has been previously published [10]. A trend toward decreased number of certain members of the phylum Firmicutes family *Clostridia* was also noted in SpA, as previously published [43, 44]. In the IBD group, certain members of Firmicutes (*Carnobacteriaceae*) were expanded, consistent with prior findings [45].

Our data on correlation between IEL-produced cytokines and the abundance of specific bacterial populations in the setting of IBD and axSpA provides an important link between functional mucosal consequences with changes in gut microbiome. In reviewing the strongest correlation, we note that in patients with CD, strong negative correlation was observed between *Fusobacteriales* and TNF-α. Prior research suggests that phylum Fusobacteria is increased in active CD [46]. Decreased IEL secretion of TNF-α in the setting of increased Fusobacteria and active inflammation could constitute a protective response. In patients with UC, we identified positive correlation between different families within *Firmicutes* (*Chistensenellaceae*) and *Proteobacteria* (*Pseudomonadaceae*) and increased IEL secretion of IL-1β. Prior studies have suggest increased abundance of *Firmicutes* and *Proteobacteria* in active IBD [45]. One mechanism by which these bacteria may be pro-inflammatory in UC is through increased IEL secretion of IL-1β. Finally, in patients with axSpA, we identified both positive and negative correlation between specific species within *Firmicutes* and IEL production of TNF-α. Others have noted increased *Prevotella* species and decreased *Bacteroides* species in axSpA [47]. The new finding that *Firmicutes* species may be linked to pro-inflammatory cytokine secretion is an area for further investigation. Clearly, additional study is required to understand these complicated relationships between IEL-produced cytokines and microbiota.

We acknowledge several limitations to this study: (1) the small number of patients only allows evaluation of changes with large effect sizes; (2) we were not powered to account for confounders, such as medications other than TNFi. TNFi use among 50% of the patients with CD and 83% of patients with axSpA compared to none of the HC and patients with UC is a major limitation to this study as is the use of steroids among three study subjects. Ideally we would have enrolled subjects with new-onset, untreated axSpA and IBD. However, this would have severely limited our enrollment of subjects and the feasibility of this study. Nevertheless, when looking at cytokine production by IELs from those subjects on TNFi or steroids compared to those not on these medications (Figs. 1 and 2), there is no obvious visual difference. This suggests that these medications were unlikely to have biased our data; (3) while microbiome data was collected via rectal swab instead of directly via mucosal biopsy, prior studies have shown this mainly affects diversity, not OTU abundance [48]; (4) the change in our cytokine expression was small; however, if each pinch biopsy is approximately 4mm^2^ and the average colon is 2m^2^ in surface area then an increase in 1 unit of secretion represents greater than 30,000-fold increase in expression of that cytokine in the colon as a whole. Some subjects displayed high IEL production of certain cytokines; however, subjects producing high levels of one cytokine were not the same for other cytokines. The presence of cytokine secretion was not driven by outliers, as data for TNF-α and IFN-γ graphed on a logarithmic axis demonstrate the full range of values; (5) histologic evidence of disease activity was not assessed when subjects lacked symptoms such as change in bowel function, but all subjects lacked visible evidence of mucosal inflammation; (6) finally, the observational study design did not permit evaluation of causal links between alterations in microbiome and IEL function.

In spite of these limitations, our data provide provocative insights into how the intestinal microbiome interacts with mucosal immune cells to contribute to disease pathogenesis within human subjects. Our findings demonstrate a complex relationship between IEL-produced cytokines and microbial diversity.

## Conclusion

The findings of this study indicate differences in IEL function among subjects with axSpA, CD, and UC compared to healthy controls. Our findings demonstrate unique correlation between altered microbiota and IEL function in these distinct populations. We believe the unique interactions between microbiota and IEL function by disease state is relevant to the pathogenesis of disease. Additional research is needed to validate these findings, yet our results give credence to the hypothesis that the gut microbiome is an important mediator in the development of IBD and axSpA.

## Additional files

Additional file 1 Supplementary Data. (DOCX 22 kb)

Additional file 2: Supplementary **Figures S1-S3**. (PDF 11177 kb)

## Abbreviations

ASAS: Assessment of SpondyloArthritis International Society
axSpA: Axial spondyloarthritis
BASDAI: Bath Ankylosing Spondylitis Disease Activity Index
CD: Crohn’s disease
CRP: C-reactive protein
ELISA: Enzyme-linked immunosorbent assay
IBD: Inflammatory bowel disease
IELs: Intraepithelial lymphocytes
IFN: Interferon
OTUs: Operational taxonomic units
PBS: Phosphate-buffered saline
SCCAI: Simple Clinical Colitis Activity Index
SpA: Spondyloarthritis
TCR: T cell receptor
TNFi: TNF inhibitor
UC: Ulcerative colitis
WBC: White blood cell
