# Supplementary material for: Functional intraepithelial lymphocyte changes in inflammatory bowel disease and spondyloarthritis have disease specific correlations with intestinal microbiota
Source: Arthritis Res Ther. 2018 Jul 20;20:149. doi: 10.1186/s13075-018-1639-3 (PMC6053728; doi:10.1186/s13075-018-1639-3)
Supplement: Supplementary file 1 — Supplementary Data. (DOCX 22 kb) [file 13075_2018_1639_MOESM1_ESM.docx]

**Table S1 Correlations between IEL number and disease activity indices.**

|  |  | **Spearman’s Rho** | *p* **value** |
| --- | --- | --- | --- |
| **axSpA subjects (n=6) IEL Number** | **Bath AS Disease Activity Index** | -0.1160 | 0.8268 |
| **Crohn’s subjects (n=10) IEL Number** | **Harvey Bradshaw Index** | -0.2963 | 0.4057 |
| **Ulcerative Collitis subjects (n=7) IEL Number** | **Simple Clinical Colitis Activity Index** | 0.5379 | 0.2130 |

Data are Rho and *p* values for spearman’s rank correlations on total IEL number and disease severity indices for their respective groups.

**Table S2 Tests of association between subject characteristics and IEL produced cytokines.**

|  |  | **TNF-α** | **IL-6** | **IL-10** | **IFN-γ** | **IL-22** | **IL-17** | **Il-1 β** |
| --- | --- | --- | --- | --- | --- | --- | --- | --- |
| **CRP** | *p* **value** | 0.071 | 0.495 | 0.122 | 0.416 | 0.59 | 0.785 | 0.122 |
|  | **β** | -.00084 | .00012 | .00728 | -.36307 | .00041 | -.00039 | .00000205 |
|  | **95% CI** | -.00314, .00146 | -.00023, .00047 | -.00205, .01662 | -1.2583, .53219 | -.00114, .00198 | -.00331, .00252 | -.0000005, .00000468 |
| **WBC Count** | *p* **value** | 0.902 | 0.909 | **0.025** | 0.624 | 0.594 | 0.974 | **0.018** |
|  | **β** | -.00054 | .00007 | .0398408 | .84209 | .00159 | -.00017 | .0000118 |
|  | **95% CI** | -.00945, .00836 | -.00128, .00144 | .00539, .07428 | -2.614, 4.2986 | -.00442, .00760 | -.01139, .01103 | .00000215, .0000214 |
| **Disease Duration (months)** | *p* **value** | 0.57 | 0.328 | 0.289 | 0.776 | 0.794 | 0.982 | 0.344 |
|  | **β** | -.00008 | -.00002 | -.00071 | -.01781 | -.00002 | -.000004 | -.00000017 |
|  | **95% CI** | -.0004, .00023 | -.00007, .00002 | -.00205, .00064 | -.1462, .11063 | -.00018, .00014 | -.00041, .00040 | -.0000006,  .0000002 |
| **Age in years** | *p* **value** | 0.121 | 0.077 | 0.216 | 0.708 | 0.8 | 0.739 | 0.297 |
|  | **β** | -.00143 | -.00025 | -.00480 | -.13721 | -.00016 | -.00039 | -.0000011 |
|  | **95% CI** | -.00326, .00039 | -.00052, .00002 | -.01253, .00293 | -.87453, .60009 | -.00145, .00112 | -.00278, .00199 | -.0000033,  .000001 |
| **Bath AS Disease Activity Index** | *p* **value** | 0.739 | 0.224 | 0.961 | 0.084 | 0.975 | 0.066 | 0.96 |
|  | **β** | .00756 | -.00167 | .00718 | 2.4161 | .00046 | .00277 | .00000207 |
|  | **95% CI** | -.05113, .06626 | -.00491, .00156 | -.37867, .39304 | -.51117, 5.3434 | -.03845, .03938 | -.00029 .00585 | -.00011, .00011 |
| **Harvey Bradshaw Index** | *p* **value** | 0.224 | 0.627 | 0.28 | 0.221 | 0.106 | 0.214 | 0.209 |
|  | **β** | -.02331 | -.00169 | -.0167 | -12.504 | .0072 | -.03911 | -.00000097 |
|  | **95% CI** | -.06409, .01746 | -.0094, .00602 | -.05016, .01661 | -34.225, 9.2177 | -.00192, .01633 | -.10593 .02770 | -.0000026, .000000671 |
| **Simple Clinical Colitis Activity Index** | *p* **value** | 0.598 | **0.001** | 0.599 | 0.414 | 0.958 | 0.564 | 0.560 |
|  | **β** | -.00187 | .00759 | -.0056 | -.0843 | .00055 | -.0051 | -.0000174 |
|  | **95% CI** | -.0104, .0067 | .0045, .0106 | -.0312, .0201 | -.3275, .1588 | -.0251, .0262 | -.0263, .0161 | -.0000089,  .00000544 |
| **Gender** | *p* **value** | 0.425 | 0.933 | 0.366 | 0.273 | 0.742 | 0.896 | 0.34 |
|  | **β** | .017817 | .00028 | .08391 | 9.483 | .00502 | .00369 | .0000249 |
|  | **95% CI** | -.02701, .06265 | -.00665, .00723 | -.10224, .27007 | -7.8111, 26.779 | -.02566, .0357 | -.05324, .06062 | -.000027, .000077 |
| **Race** | *p* **value** | 0.695 | 0.708 | 0.831 | 0.828 | 0.298 | 0.779 | 0.945 |
|  | **β** | -.02712 | -.00398 | -.06129 | -5.8370 | .04856 | -.02437 | .00000556 |
|  | **95% CI** | -.16628, .11202 | -.02534, .01738 | -.64151, .51893 | -60.042, 48.368 | -.0446 .1418 | -.19969, .15095 | -.00015, .00016 |
| **Fam. Hist. of autoimmunity** | *p* **value** | 0.186 | 0.29 | 0.214 | 0.485 | 0.206 | 0.331 | 0.153 |
|  | **β** | .03129 | .00386 | -.12272 | 6.4841 | -.02033 | .02909 | -.0000395 |
|  | **95% CI** | -.01580, .078402 | -.00343, .01116 | -.31939, .07394 | -12.173, 25.142 | -.05239, .01173 | -.03088, .08907 | -.0000944, .0000154 |
| **Smoking (yes/no)** | *p* **value** | **0.008** | 0.507 | 0.658 | 0.07 | 0.101 | 0.051 | 0.483 |
|  | **β** | .06068 | -.00243 | .04414 | -16.491 | .02619 | -.05716 | .0000196 |
|  | **95% CI** | .10428, .01709 | -.00980, .00493 | -.15641, .24470 | -34.408, 1.427 | -.00536, .05774, | -.11471, .00039 | -.0000366, .0000758 |
| **Use of TNF-α inhibitor** | *p* **value** | **0.001** | 0.111 | 0.055 | **0.039** | 0.372 | 0.162 | 0.111 |
|  | **β** | .07631 | .006062 | .19621 | 19.710 | -.01523 | .04383 | .0000463 |
|  | **95% CI** | .03263, .11998 | -.00147, .01359 | -.00481, .39724 | 1.0948, 38.326 | -.04942, .01895 | -.01847, .10613 | -.0000112, .0001038 |
| **Fish (d.f.=5)** | **Chi-Sq** | 4.78 | 5.501 | 7.044 | 1.917 | 2.863 | 6.814 | 2.509 |
|  | *p* **value** | 0.4433 | 0.3578 | 0.2174 | 0.8605 | 0.721 | 0.2349 | 0.7751 |
| **Whole grains (d.f.=4)** | **Chi-Sq** | 2.583 | 4.867 | 3.189 | 1.396 | 1.084 | 5.387 | 2.564 |
|  | *p* **value** | 0.6298 | 0.3012 | 0.5267 | 0.8449 | 0.8968 | 0.2499 | 0.6332 |
| **Fruits (d.f.=8)** | **Chi-Sq** | 7.403 | 6.963 | 2.587 | 7.147 | 8.443 | 7.445 | 8.092 |
|  | *p* **value** | 0.4937 | 0.5406 | 0.9576 | 0.5209 | 0.3915 | 0.4895 | 0.4245 |
| **Vegetables (d.f.=6)** | **Chi-Sq** | 10.438 | 8.132 | 1.088 | 2.416 | 11.289 | 9.194 | 10.852 |
|  | *p* **value** | 0.1074 | 0.2286 | 0.982 | 0.8777 | 0.0798 | 0.163 | 0.093 |
| **Red Meat (d.f.=6)** | **Chi-Sq** | 7.653 | 7.996 | 11.924 | 3.209 | 2.985 | 3.596 | 7.764 |
|  | *p* **value** | 0.2646 | 0.2384 | 0.0637 | 0.7821 | 0.8107 | 0.7312 | 0.2559 |

Data are *p* values, beta parameter estimates, and 95% confidence interval. *P* values, beta estimates, and parameter estimates for continuous variables were produced from univariate linear regression; chi-sq statistics produced from chi-squared analysis. Dietary intake of fish, whole grains, fruits, vegetables, and red meat collected categorically based on monthly intake.
