# Supplementary material for: Functional intraepithelial lymphocyte changes in inflammatory bowel disease and spondyloarthritis have disease specific correlations with intestinal microbiota
Source: Arthritis Res Ther. 2018 Jul 20;20:149. doi: 10.1186/s13075-018-1639-3 (PMC6053728; doi:10.1186/s13075-018-1639-3)
Supplement: Supplementary file 2 — Supplementary Figures S1-S3. (PDF 11177 kb) [file 13075_2018_1639_MOESM2_ESM.pdf]

Supplementary Figure 1

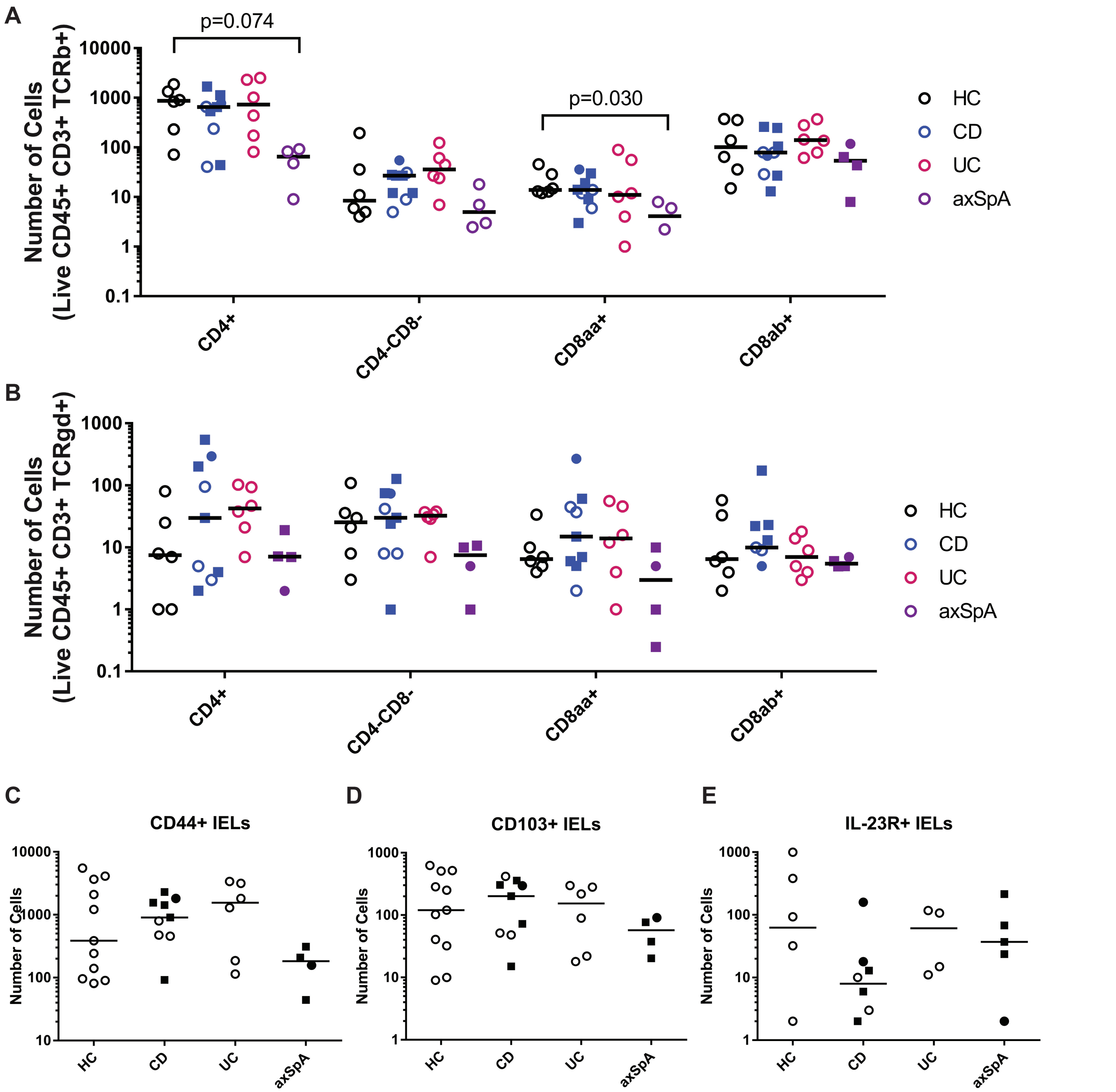

Supplementary Figure 1. IEL characteristics in subject groups. IELs were obtained from colon biopsies and evaluated by flow cytometry as described in Methods. (A) Subgroups of TCRβ+ and (B) TCRγδ+ IELs are shown as well as the number of (C) CD44+, (D) CD103+, and (E) IL-23R+ IELs. Each dot represents a case/control and bars are the median. A solid square indicates subjects on a TNF inhibitor, a solid dot indicates the subject was taking steroids, and an open dot indicates the subject was taking neither. Kruskal-Wallis with Dunn's post-hoc analysis was used to determine statistical significance; p-values are as noted on the graphs.

Supplementary Figure 2

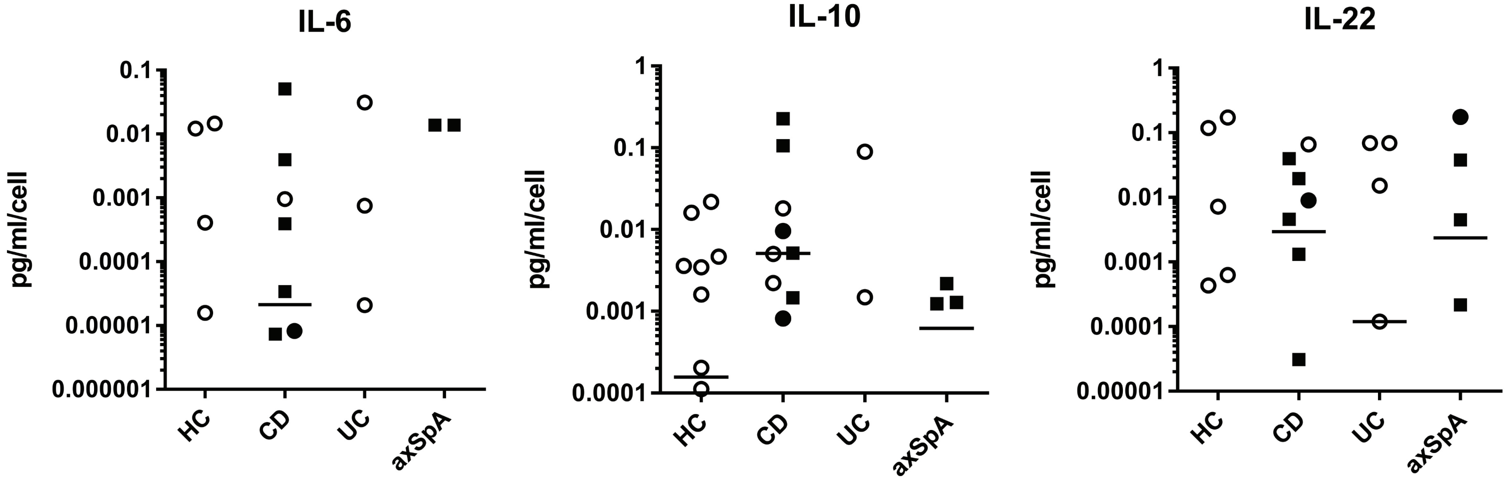

Supplementary Figure 2. IEL produced cytokines in study groups. Colon IELs were mitogen-stimulated overnight and secreted cytokines measured by ELISA. IL-6, IL-10, and IL-22 in pg/ml was normalized to the number of IELs collected from each subject. Each dot represents a case/control analyzed. A solid square indicates subjects on a TNF inhibitor while a solid dot indicates the subject was taking steroids; an open circle indicates the subject was taking neither medication. Subjects with undetectable cytokine levels are absent from the graphs due to the logarithmic scale. Statistical differences were not found by Kruskal-Wallis with Dunn's post-hoc analysis for pairwise differences.

Supplementary Figure 3

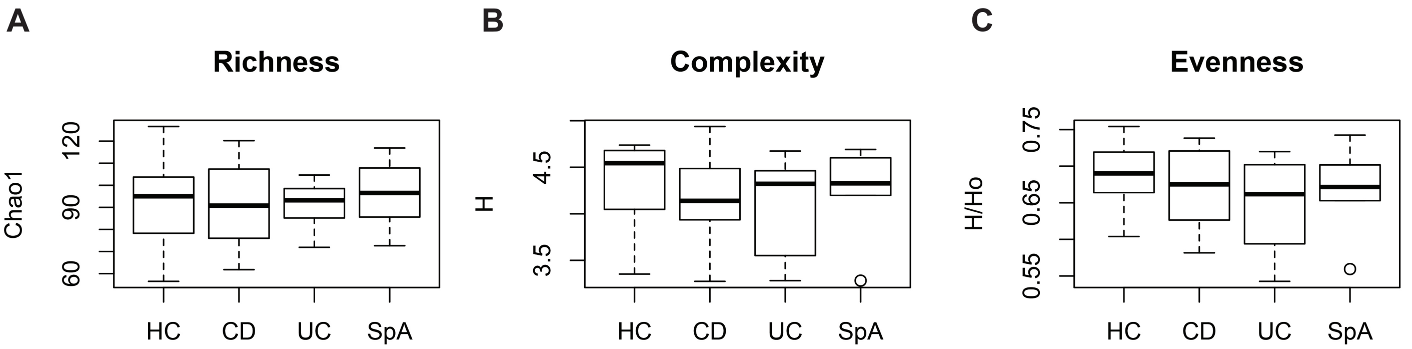

Supplementary Figure 3. Alpha-diversity measures for richness (Chao1), complexity (ShannonH), and evenness in subject groups are shown as box-and-whisker plots. Statistical analysis performed with one-way ANOVA failed to reveal statistical significance.
